# On interevent time distributions of avalanche dynamics

**DOI:** 10.1038/s41598-019-56764-6

**Published:** 2020-01-17

**Authors:** Pinaki Kumar, Evangelos Korkolis, Roberto Benzi, Dmitry Denisov, André Niemeijer, Peter Schall, Federico Toschi, Jeannot Trampert

**Affiliations:** 1 Department of Applied Physics, Eindhoven University of Technology, P.O. Box 513, 5600 MB Eindhoven, The Netherlands; 2 Department of Earth Sciences, Utrecht University, P.O. Box 80115, 3508 TC Utrecht, The Netherlands; 3 Dip. di Fisica and INFN, Università “Tor Vergata”, Via della Ricerca Scientifica 1, I-00133 Roma, Italy; 4 Institute of Physics, University of Amsterdam, 1098 XH Amsterdam, The Netherlands; 5 Department of Mathematics and Computer Science, Eindhoven University of Technology, P.O. Box 513, 5600 MB Eindhoven, The Netherlands; 6 Istituto per le Applicazioni del Calcolo, Consiglio Nazionale delle Ricerche, Via dei Taurini 19, 00185 Rome, Italy

**Keywords:** Statistical physics, Structure of solids and liquids

## Abstract

Physical systems characterized by stick-slip dynamics often display avalanches. Regardless of the diversity of their microscopic structure, these systems are governed by a power-law distribution of avalanche size and duration. Here we focus on the interevent times between avalanches and show that, unlike their distributions of size and duration, the interevent time distributions are able to distinguish different mechanical states of the system. We use experiments on granular systems and numerical simulations of emulsions to show that systems having the same probability distribution for avalanche size and duration can have different interevent time distributions. Remarkably, these interevent time distributions look similar to those for earthquakes and, if different from an exponential, are indirect evidence of non trivial space-time correlations among avalanches. Our results therefore indicate that interevent time statistics are essential to characterise the dynamics of avalanches.

## Introduction

Many physical systems subject to small external driving forces exhibit complex burst dynamics in space and in time^1–7^. Burst events are the signature of energy release in the system and, in many cases, they have successfully been described in terms of avalanche dynamics. Both theoretically and experimentally, the statistical distribution of the size, $S$, and the time duration, ${t}_{E}$, of avalanches have been shown to satisfy well-defined scaling laws. In the case of plasticity of soft glasses and fracture dynamics of amorphous solids, the scaling exponents are mostly independent of the details of the microscopic interactions, suggesting some form of universality^6,8–11,12,13^, although the exponents from experiments vary within about 10%^10,14^. Theoretically, the scaling exponents have been explained by a number of different mean- and non mean-field theories, which take into account the basic physical properties of the system and its intrinsic “randomness”^2,8,10^. This apparent universality might also explain the observed scaling properties seen in earthquake dynamics, e.g. the well-known Gutenberg-Richter law^6,8,15^. The question naturally arises whether some statistical properties of avalanches are able to discriminate between different states of these systems. One interesting quantity is the interevent time, ${t}_{i}$, (also referred to as recurrence time, return time or waiting time) between two consecutive avalanches^7^. Most of our information on the interevent time distribution $P({t}_{i})$ comes from the analysis of earthquake catalogs. There is a general consensus for earthquakes, that at short time scales $P({t}_{i})\sim \mathrm{1/}{t}_{i}$ follows Omori’s law^16^, while at very long time scales $P({t}_{i})\sim exp(\,-\,\alpha {t}_{i})$. The exponential behaviour is explained by the (reasonable) assumption, that for long time scales, earthquake events are likely to be independent. There is however an intermediate time scale, and Corral^17^ showed that $P({t}_{i})$ is in general better fitted by a Gamma distribution, leading to a time scale defined by the average interevent time. Interestingly, this Gamma distribution is observed for many different geographic regions, as well as for the whole Earth, once $P({t}_{i})$ is re-scaled to the regional or global average interevent time^17^. A detailed investigation of seismicity induced by mining and fluid injection revealed the same Gamma distribution for interevent times^18,19^ as well as for acoustic emissions of various other systems^20,21^. Within seismology, there is however no consensus on the necessity of such an intermediate time scale to explain the observations. Generally, if the interevent time distribution is independent of any size threshold or region, the only possible shape for $P({t}_{i})$ is exponential, unless complex space and time correlations are present and the system is close to criticality^22,23^. Another option is that the Gamma distribution emerges as a superposition of independent probability distributions, one with an exponential tail and one with the Omori short-time behaviour^24,25^. In the case of superposition, $P({t}_{i})$ does not reveal any new physics besides the well documented avalanche scaling laws (Gutenberg-Richter and Omori). Finite detection thresholds^26^ have also been suggested to explain the emergence of Gamma distributions. In the past, studies have tried to settle this question by analyzing the fit of data to various functional forms for $P({t}_{i})$. Without the knowledge of the underlying state of the system, best fit arguments are obviously difficult to make. We will instead analyze systems where we control the mechanical state and investigate whether $P({t}_{i})$ informs us on physics beyond that of $P(S)$ and $P({t}_{E})$.

We report on a systematic study of the distribution of interevent times, $P({t}_{i})$, across different model systems to show that it provides crucial information on the mechanical state of the material. We complement experimental measurements on granular systems with numerical simulations on emulsion-like models, and compare the resulting interevent time distributions with those for earthquakes. Our results uniquely show that unlike the statistical properties of avalanche sizes and durations, the probability of interevent times $P({t}_{i})$ strongly depends on the material properties. For relatively low normal stresses applied during the experiments or a low rigidity for the numerical system, the interevent time distribution is exponential with an Omori behaviour at small time scales. For high normal stresses and rigidity, $P({t}_{i})$ is Gamma distributed and similar to that reported for earthquakes at long time scales^17^, with an Omori scaling at small time scales. This implies that the functional shape of $P({t}_{i})$ depends on the mechanical properties of the system and that, in addition to avalanche size and duration, it provides a crucial measure to distinguish avalanche-like relaxation mechanisms. We show that spatio-temporal correlations are responsible for these different regimes, and therefore interevent time distributions provide insight into the nature of correlated deformations in dense suspensions and earthquakes.

## Results

**Figure 1:**
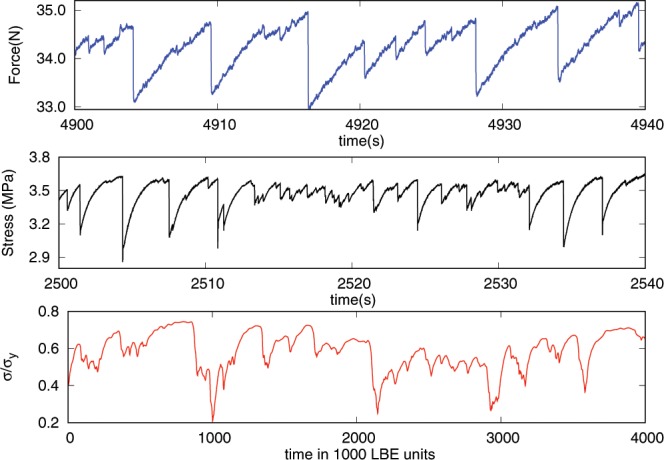
Snapshot of the stress/force-time evolution for the different systems. Top: sample from the shear cell, middle: sample from the rotary shear experiment and bottom: results from an LBE simulation.

We use granular systems under well-controlled normal stresses, apply slow shear strain rates to induce avalanches and monitor them with high temporal resolution. The recorded force signal from our shear cell (see Methods section) exhibits strong intermittency: force increases are followed by sudden force drops that demarcate energy release events (Fig. 1 top). We measure the force drops with high temporal resolution to resolve the dynamics of both the large and small avalanche events. Previous experiments have shown that the applied shear strain rate is sufficiently slow to separate individual avalanches and avoid avalanche overlap^27^. This enables us to extract a wide range of predicted scaling exponents and scaling functions that identify the underlying slip statistics and dynamics^11,27^. The second granular system, a rotary shear cell (see Methods section), similarly shows strong intermittency: stress slowly increases, followed by sudden stress drops that correspond to acoustic emission (AE) events (Fig. 1 middle). We monitor the AEs with piezoelectric transducers at a high rate to infer the scaling laws. We also analyse numerical simulations of a two-dimensional emulsion using Lattice Boltzmann Equation (LBE) modelling^28^ (see Methods section). The model aims at simulating two repelling fluids. Coarsening is strongly suppressed by using a frustration mechanism, which stabilises the interface. The system exhibits a yield stress rheology with a non-Newtonian relation between stress and shear strain rate above the yield stress^29^. For a small imposed shear strain rate, such that the stress is below yield stress, a clear stick-slip behaviour is observed (Fig. 1 bottom).

### Avalanche size distributions

**Figure 2:**
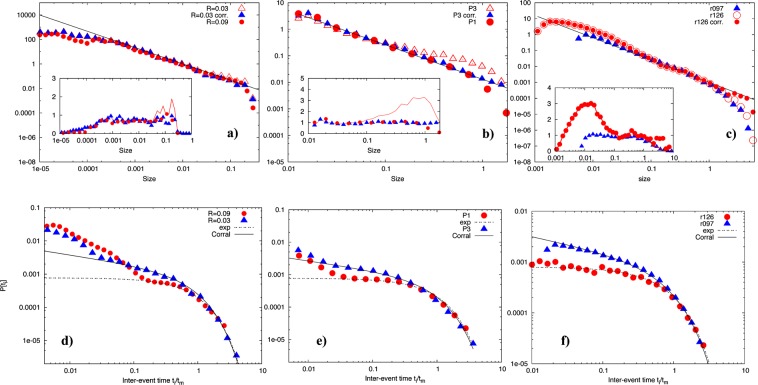
Scaling properties of avalanche sizes measured for various systems (**a**–**c**). Red and blue symbols correspond to $P({S}_{i})$ for low and high material parameter settings. The black lines correspond to slopes of −4/3 for LBE (**a**), −1.3 for the shear cell (**b**) and −1.4 for the rotary cell (**c**). Open symbols correspond to distributions with bumps. Where present, they have been modelled using $P({S}_{i})\,\exp (\,-\,b{S}_{i}+c{S}_{i}^{2})$ (filled symbols). The inset shows the collapse of the distributions onto $P({S}_{i}){S}_{i}^{\tau }$, symbols for distributions without bumps and lines for distributions with bumps. The latter are then collapsed onto $P({S}_{i}){S}_{i}^{\tau }\,\exp (\,-\,b{S}_{i}+c{S}_{i}^{2})$, with the same $\tau$ (symbols). Scaling properties of interevent times measured for various systems (**d**–**f**). Red symbols correspond to cases of low rigidity or normal stresses and follow an exponential distribution. Blue symbols correspond to cases of high rigidity or normal stresses and follow a Gamma distribution. The shear cell experiment P1 is conducted at a normal stress of 4 kPa and P3 at 9.6 kPa. The rotary shear experiment r126 is conducted at a normal stress of 2 MPa and r097 at 8 MPa.

The scaling properties of the avalanche sizes are similar for all our systems (Fig. 2a–c). For each experiment, results correspond to two different material settings. The distributions clearly show a scaling with a best fit exponent $\tau$ between 1.3–1.4 over 2–3 orders of magnitude in avalanche size, irrespective of their material properties. We refrain from giving an explicit uncertainty on the exponents, but they are in the range of previous studies^10,14^. Some distributions show bumps for larger avalanche sizes, which have been described as corresponding to systems near criticality^30^. We can reduce those using $P({S}_{i})=\frac{a}{{S}_{i}^{\tau }}\,\exp (b{S}_{i}-c{S}_{i}^{2})$^30^, without affecting the exponent of the power law. The inset shows the collapse of the distributions underlining our claim that the size distributions portray a power law over several orders of magnitude with an exponent close to −4/3. Their exponents also agree with those for earthquakes^8,31^.

### Avalanche interevent time distributions

**Figure 3:**
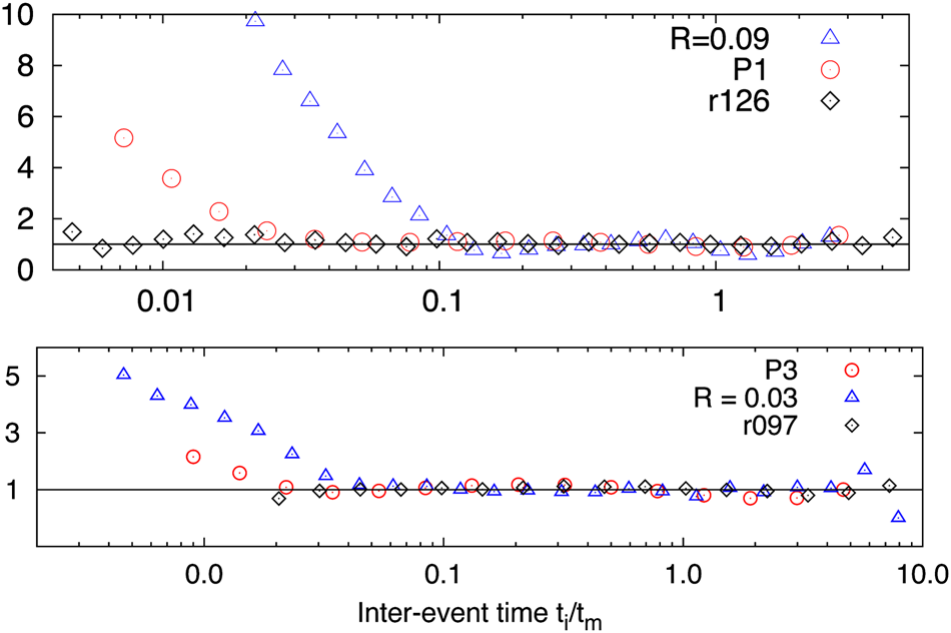
Ratio $R({t}_{i})$ between the probability distributions $P({t}_{i})$ plotted in Fig. 2d–f and the one given by Eq. (1) with $\gamma =0.7$ for the blue triangles (bottom panel) and $\gamma =1.0$ for those corresponding to the red dots (top panel).

The scaling properties of interevent times, however, behave markedly differently depending on the material settings in the experiments. For large packing fraction or normal stress, LBE simulation R = 0.03, shear cell experiment P3 and rotary experiment r097, all data follow a Gamma distribution (Fig. 2d–f) given by

1 $$G({t}_{i})=\frac{C}{{t}_{m}}{(\frac{{t}_{i}}{{t}_{m}})}^{(\gamma -1)}\exp (\,-\frac{{t}_{i}}{{t}_{m}})$$

with $\gamma =0.7$, ${t}_{m}=\langle {t}_{i}\rangle$ the average interevent time and *C* a normalization constant. This is the same Gamma distribution as that reported for earthquakes^17^. For relatively small packing ratio or normal stress data corresponding to LBE simulation R = 0.09, shear cell experiment P1 and rotary experiment r126, $P({t}_{i})$ behaves markedly differently (Fig. 2d–f). For long ${t}_{i}/{t}_{m}$, the systems show an exponential behaviour, corresponding to $\gamma =1$ in Eq. (1), clearly distinct from those following the Gamma distribution. The short time behaviour $P({t}_{i})\sim \mathrm{1/}{t}_{i}$ consistent with Omori’s law is observed in all cases, except the rotary experiments, but no data collapse is obtained, i.e. the time scale separation between Omori’s and the exponential or Gamma behaviour is expressed differently depending on the physical system. The quality of the fitting is clearer from Fig. 3, where we plot the ratio $R({t}_{i})\equiv P({t}_{i})/G({t}_{i})$ where $P({t}_{i})$ are the different probability distributions shown in the Fig. 2d–f and $G({t}_{i})$ is given by Eq. (1).

Figure 2 demonstrates the main point of our paper: $P({t}_{i})$ changes upon varying the system properties, although no major change is observed in the avalanche size distribution. The shape of $P({t}_{i})$ does not depend on the threshold values that were used to acquire and denoise the data (see Methods section). This is further evidenced below where we show an example of $P({t}_{i})$ conditioned on size to identify memory effects in the system. Rather, since the interevent times inform us on the relaxation time of the system, and their distribution on the relaxation process, a change of $P({t}_{i})$ indicates a fundamental change in the nature of the underlying relaxation processes.

## Discussion

From the above, we can draw some general and nontrivial conclusions.

The statistical properties of avalanche size, $S$, and duration, ${t}_{E}$ (a detailed analysis for LBE is shown in the Methods section; we do not repeat the analysis for the shear cell, which is shown elsewhere^11^; concerning the rotary shear experiments, our recording system of AEs does not give us direct access to avalanche durations) are independent of the system material properties. Physically this means that, once the system starts to release elastic energy, it does this independently of any material constants. This is one of the basic assumptions in many theoretical frameworks so far proposed to predict the (universal) scaling properties of avalanche size distribution.

The interevent time distributions, however, show a clear dependency on system properties. They also display a clear signature of two different time scales shaping their probability distribution. At short time scales, although not the focus of this report, we observe events clustered in time where the interevent time distribution is $P({t}_{i})\sim \mathrm{1/}{t}_{i}$, regardless of material property. This is consistent with Omori’s law observed for earthquakes, and is most likely due to smaller size events triggered by some master event (after-shocks). Note, however, that our analysis is independent of any definition of main- and after-shock. At longer time scales, the interevent time distribution is given by Eq. (1), where the exponent $\gamma$ depends clearly on material properties. $\gamma$ changes from close to 1 to about 0.7, a value often quoted for earthquakes^17^.

The underlying question is which system parameter is driving this change in $P({t}_{i})$. For our LBE experiment, we quoted $r$, which can readily be related to packing fraction, material stiffness or rigidity (see Methods section). Concerning the analogue experiments, which are distinguished by changing the normal stresses applied during the experiments, it is not as straight forward the relate the normal stresses to packing fraction as it changes during the experiment. Force chains have also been advocated as being responsible for phenomena observed in granular media^32–34^, but just as with packing fraction, we cannot easily translate confining pressure or R into force chains. Maybe rigidity of the system is simply the underlying fundamental parameter, but without a corresponding theory all these remain possibilities. It has also been suggested to study the full temporal shape of avalanches^11,35,36^. While also important, this is clearly beyond the scope of our report. We are not aware of any theoretical framework able to describe these features and/or explain how $P({t}_{i})$ changes with material properties. It has been argued^23^ that for Eq. (1) to emerge with $\gamma \ne 1$, correlations have to be present in the system. The question thus remains whether our systems display any correlations.

**Figure 4:**
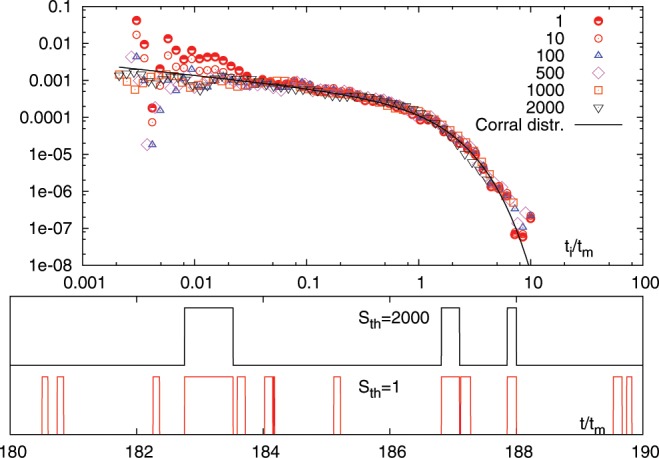
Top panel: $P({t}_{i}/{t}_{m},{S}_{th})$ for ${S}_{th}\in [1,2000]$. In the range ${S}_{th}\in [1,2000]$ the probability distributions $P({t}_{i}/{t}_{m},{S}_{th})$ collapse on the same master curve for $t/{t}_{m}\ge 0.03$, which is close to the Gamma distribution with $\gamma =0.7$. Bottom panels: event snapshots for the two extreme cases ${S}_{th}=1$ and ${S}_{th}=2000$. The former displays indeed many events also seen in the latter.

To investigate whether or not any time correlations exist (i.e. “memory effects” in the avalanche dynamics), we follow Corral^37^ and consider the probability density of the interevent times conditioned on the avalanche size. We focus on the numerical simulation for ($R=0.03$) and define $P({t}_{i}/{t}_{m},{S}_{th})$ as the interevent time probability density for a subset of events with size $S > {S}_{th}$. Upon increasing ${S}_{th}$, if $P({t}_{i}/{t}_{m},{S}_{th})$ is not exponentially distributed and it does not change its shape, then time correlations (or memory effects) exist between the interevent times for different threshold values ${S}_{th}$. For a process with no correlations, one can show that the only scale invariant distribution is the exponential distribution^22^. On the other hand, correlations introduce new invariant functions in the process. The robust shape of the distributions for different ${S}_{th}$ in Fig. 4 in this context clearly demonstrates that the interevent time is indeed correlated in a non-trivial way with the size of the previous event.

Motivated by this observation, we further looked for spatio-temporal correlations among avalanche events by analyzing sub-regions of the system. We consider two disjoint regions, say $A$ and $B$, and their set union, $A\cup B$ including both regions, over the full simulation time interval. If the regions are independent, the only possible interevent time distribution is exponential^22^. If there are space-time correlations between the different regions, then we expect that the probability distributions $P({t}_{i}/{t}_{m}(A))$, $P({t}_{i}/{t}_{m}(B))$ and $P({t}_{i}/{t}_{m}(A\cup B))$ are all the same and none of them is exponential^23,37^. If at least in one of the regions $A$, $B$ and $A\cup B$ the interevent time is exponentially distributed, then we can argue that the Gamma distribution observed for the whole system is just by accident. This constitutes a severe test to uncover (although indirectly) space-time correlations in the system.

**Figure 5:**
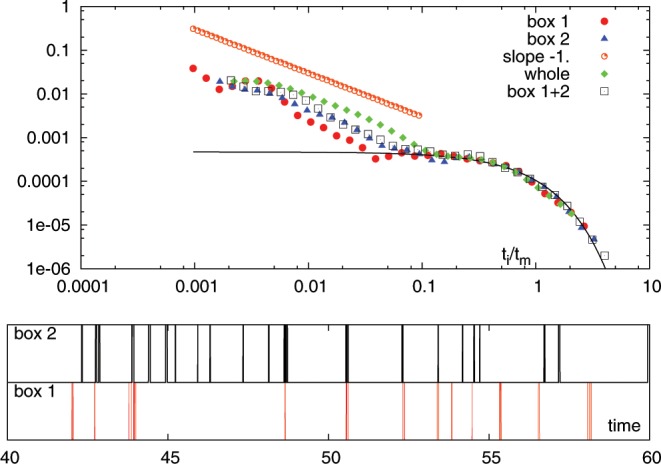
Top panel: $P({t}_{i}/{t}_{m})$ for the different boxes, their sum and the whole system for the case $R=0.09$. Bottom panels: event snapshots showing the independence of the 2 time series.

**Figure 6:**
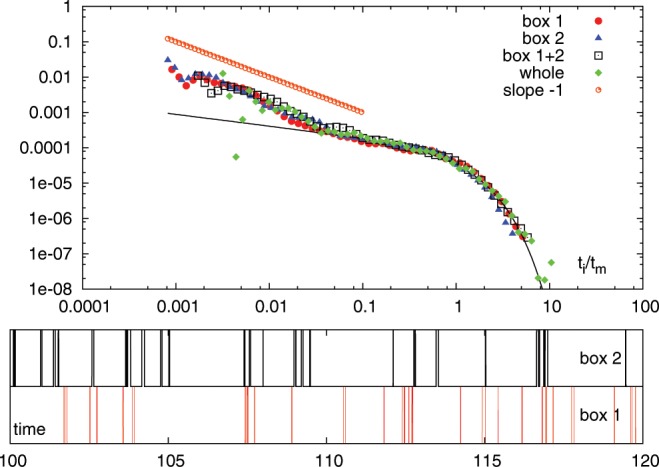
Top panel: $P({t}_{i}/{t}_{m})$ for the different boxes, their sum and the whole system for the case $R=0.03$. Bottom panels: event snapshots. Bottom panels: event snapshots showing the independence of the 2 time series.

In the original picture of avalanche dynamics in amorphous systems, the usual assumption is that while the avalanches themselves are highly correlated events, they occur at random uncorrelated times i.e. with an exponential distribution for interevent times $P({t}_{i}/{t}_{m})$. For such uncorrelated random events, we expect therefore that the interevent time distributions ${t}_{i}/{t}_{m}(A)$ and ${t}_{i}/{t}_{m}(B)$ are both also exponentially distributed. This is what happens for our LBE simulation at ($R=0.09$) as shown in the upper panel of Fig. 5. All curves follow the exponential distribution. In the lower panel of the same figure we show a snapshot of the time series illustrating the avalanche events in the two regions (referred to as *box* 1 and *box* 2). In the upper panel we also show the interevent time distribution for the whole system, which, as we know, is also exponential (Fig. 2d–f). Note that the curves do not collapse, as they apparently sense the short time scale differently. The same analysis for $R=0.03$) gives a different picture (Fig. 6). In all cases, we indeed observe the same probability distribution for $P({t}_{i}/{t}_{m})$, which is not exponential. As we have argued above, this can only be true if there are non-trivial correlations and/or memory effects between the two different regions. Note that in this case the curves collapse also for the short Omori time scales.

While it is hard to identify the exact nature of such correlations, a clear picture emerges from our observations. Overall our results demonstrate that the interevent time distribution $P({t}_{i})$ is able to disentangle the statistical properties of systems at different material states, whereas this is not possible by looking only at the avalanche size distribution and duration. As the interevent times are directly related to the relaxation time of the system, their distribution should contain information about the relaxation mechanism. We thus conclude that the different interevent time distributions we observe, and their interpretation in terms of absence and presence of memory effects, indicate fundamentally different relaxation mechanisms for our various systems.

**Figure 7:**
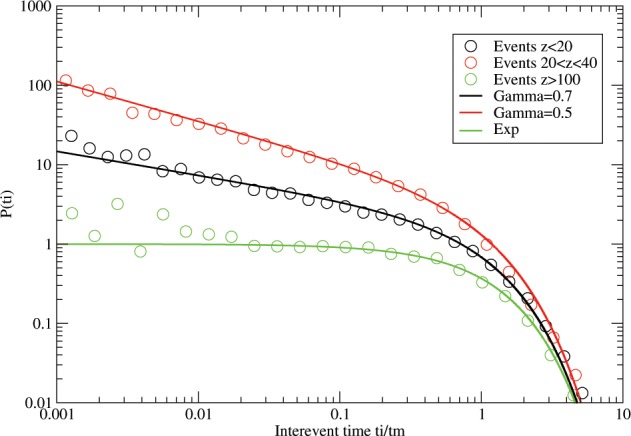
Interevent times for earthquakes from the revised global ISC catalogue covering the years 1904–2015 (10.31905/D808B825).

We have mentioned above that earthquake size distributions follow a power law compatible with our systems^8,14,31^. Earthquake interevent times for certain regions or the whole Earth have been observed to follow a Gamma distribution with $\gamma =0.7$^17^. We reanalyzed the latest revised global ISC event catalogue, where we split the crustal events into 2 sets of roughly equal size and one mantle set (Fig. 7). Crustal earthquakes shallower than 20 km depth follow a Gamma distribution with $\gamma =0.7$, crustal earthquakes with depths between 20–40 km follow a curve with $\gamma =0.5$. Going deeper into the crust, the lithostatic pressure increases and so does the rigidity of the brittle crustal material. This suggests that the slope of the intermediate regime gets steeper as the rigidity of the material increases. Surprisingly, for earthquakes below the crust and deeper than 100 km, we see an exponential behaviour. Here the material is thought to be more ductile (lower rigidity), despite a higher lithostatic pressure, and events are apparently generated randomly. Furthermore, we ignored events with a magnitude lower than 5.5 eliminating most aftershocks. Therefore, the Gamma distribution cannot be generated by an interplay of Omori and exponential behaviour as previously suggested^24,25^. In conclusion, our results from laboratory experiments and simulations clearly give new insight into the origin of these interevent time distributions.

## Methods

### Avalanche definitions

An avalanche is defined as the event during which the system experiences a stress drop that results in the release of elastic waves (AE). This implies that the time ${t}_{s}(i)$ at which the avalanche ${A}_{i}$ starts is identified by a change of sign (from positive to negative) of $d\sigma /dt$ while the final time ${t}_{f}(i)$ is identified by a change of sign (from negative to positive) of $d\sigma /dt$. Here $\sigma$ is the space average stress of the system. The time difference ${t}_{E}(i)\equiv {t}_{f}(i)-{t}_{s}(i)$ is therefore the avalanche duration time whereas the time difference ${t}_{i+1}\equiv {t}_{s}(i+\mathrm{1)}-{t}_{f}(i)$ is the interevent time between the avalanche ${A}_{i+1}$ and ${A}_{i}$.

The avalanche size ${S}_{i}$ can be computed in several ways. In principle it should be related to the energy release by the system, i.e. ${E}_{i}=\int \,dt\,\sigma \dot{\sigma }$ where the integral is performed during the duration time ${t}_{E}(i)$ of the avalanche ${A}_{i}$. Other suitable definitions are possible. In the LBE simulations we use the avalanche size ${S}_{i}$ expressed by the integral in time of the largest displacement square^31^. In the rotary shear experiments, we use the median of the largest square acoustic amplitudes recorded on the 16 transducers during the avalanche. In the shear cell, we directly use the force drops measured by high rate force sensors^11^. Whatever definition we use for the size ${S}_{i}$, the probability distribution $P({S}_{i})$ must share the same scaling properties observed for the energy release ${E}_{i}$. The advantage of using other definitions is due to the fact that avalanches are strongly intermittent features both in space and in time. Therefore using a variable which is, by definition, tightly related to intermittency should increase the scaling region of $P({S}_{i})$ while the space average stress $\sigma$ or its time derivative $\dot{\sigma }$ tends to smear out large fluctuations and, consequently, reduce the scaling region. For LBE simulations and for the rotary experiments we did check that our definition of the size ${S}_{i}$ is consistent, scaling wise, with the scaling properties of ${E}_{i}$. For the shear cell, we use the stress drop as a measure of the ${E}_{i}$ assuming that ${E}_{i}=\int \,\sigma \,\dot{\sigma }dt\sim {\sigma }_{\ast }(\sigma ({t}_{f}(i)-\sigma ({t}_{s}(i)))$, with ${\sigma }_{\ast }$ some average value of $\sigma$ in the system. Concerning numerical simulations, a more systematic discussion using different size definitions can be found elsewhere^38^.

For our purpose, it is crucial to properly identify the initial ${t}_{s}(i)$ and final ${t}_{f}(i)$ of avalanches. In fact, while $P({S}_{i})$ is a rather robust feature of the system regardless of the definition of ${S}_{i}$, the probability distribution of the interevent time $P({t}_{i})$ strongly depends on the avalanche definition i.e. on the definition of the initial and final time of the avalanche. For shear driven systems, as in our case, the avalanche definition should be linked to stress dynamics, i.e. to the stress drops. Let’s consider the probability distribution $P({t}_{i}|{S}_{\ast })$ of interevent times ${t}_{i}$ occurring between avalanches whose sizes ${S}_{i}$ are greater or equal some threshold ${S}_{\ast }$. The quantity $P({t}_{i}|{S}_{\ast })$ is a rather non-trivial characterization of the statistical properties of the avalanche dynamics. To understand this point let us consider three avalanches of sizes ${S}_{i}$, ${S}_{i+1}$ and ${S}_{i+2}$ and corresponding to interevent times ${t}_{i+1}$ and ${t}_{i+2}$. If ${S}_{i+1} < {S}_{\ast }$ while both ${S}_{i}$ and ${S}_{i+2}$ are larger than ${S}_{\ast }$, then the two interevent times ${t}_{i+1}$ and ${t}_{i+2}$ disappear from the statistical records while the longer interevent time ${t}_{i+1}+{t}_{i+2}$ appears. Obviously, $P({t}_{i}|{S}_{\ast })$ depends on the definition of ${S}_{i}$. However, if the scaling properties of $P({S}_{i})$ are independent on the definition of avalanche size ${S}_{i}$, then we expect that this is also true, at least statistically, for $P({t}_{i}|{S}_{\ast })$. We explicitly show this in the report for the LBE simulations (Fig. 4) and checked that this is the case for the granular experiments.

### Laboratory experiments

**Figure 8:**
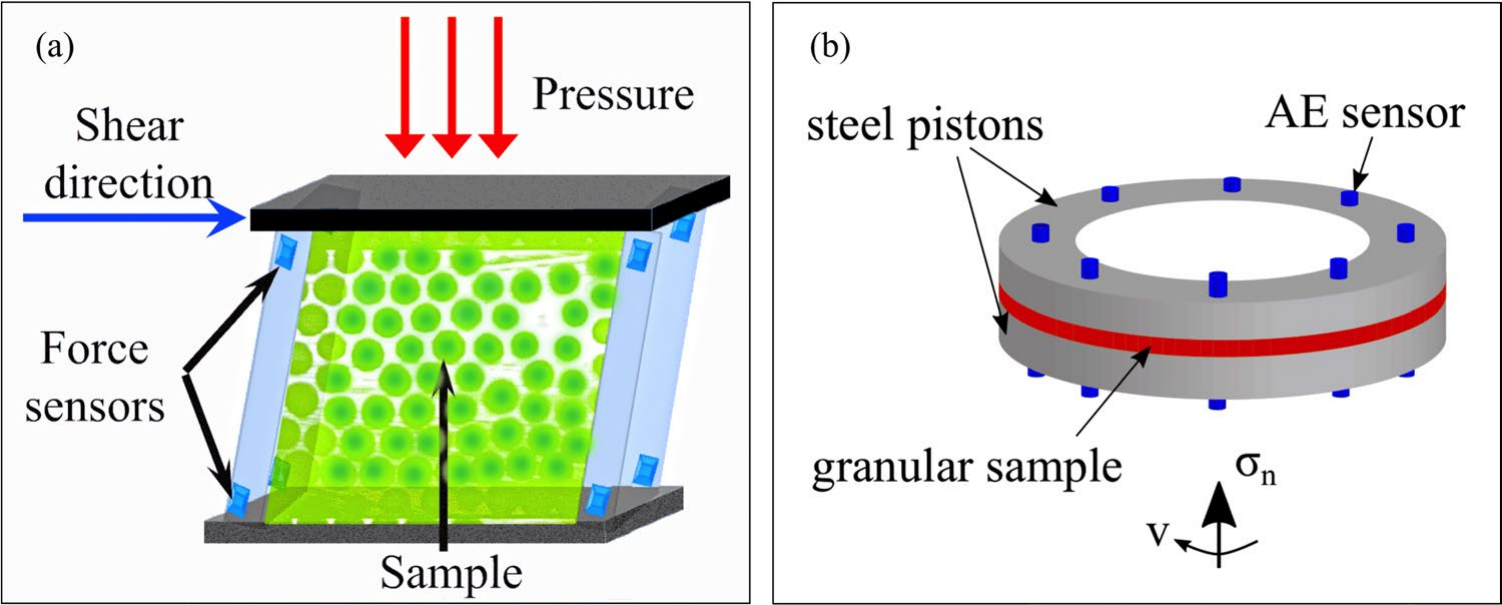
(**a**,**b**) Schematically show the granular shear cells with force or acoustic emission (AE) sensors in the walls. Loads imposed perpendicular to shear exert a constant confining pressure.

We use two different granular systems, where we apply well-controlled normal forces and slow shear strain rates (Fig. 8).

The first system, a shear cell, consists of 3 · 10^5^ polymethyl methacrylate spheres with a diameter of $d=1.5$ mm and a polydispersity of ~5%, and is deformed at a constant shear strain rate of $\dot{\gamma }=9.1\cdot {10}^{-4}\,{s}^{-1}$ and constant normal stress, using a shear cell with a confining top plate (Fig. 8a). By placing weights onto the confining plate, we vary the normal stress from 4 to 9.6 kPa, experiments P1 and P3, respectively, resulting in a packing fraction $\phi$ between 55% and 60%. We monitor shear-induced force fluctuations using force sensors included in the shearing walls. The experiment is described in detail elsewhere^11^, including protocols to guarantee adequate time resolution of the fluctuations.

The second granular system, a rotary shear cell, consists of glass bead layers that are sheared using a servo-controlled rotary shear apparatus (Fig. 8b). The average particle size is 0.5 mm and the standard deviation 0.1 mm. For each experiment, an approximately 4.5 mm thick layer of glass beads is deposited in an annular-shaped shear cell that consists of four independent steel rings: two of them act as pistons that provide vertical confinement, whereas the other two provide lateral support. The cell is then placed in the rotary shear apparatus, built inside an Instron 8862 testing machine. The servo-controlled Instron actuator is used to prescribe a constant normal stress condition (8 MPa for experiment r097 and 2 MPa for experiment r126), and a Parker MH-205 motor to maintain a constant angular velocity of 0.02°/*s* by rotating the bottom piston. An axially mounted load cell (±100 kN range, 0.008 kN resolution) measures the normal stress, and a pair of laterally mounted load-cells (each 20 kN maximum load, 0.008 kN resolution) the traction. The experiments are started at a random close packing although there is an increase over time due to the breaking of particles. Avalanches are defined by stress drops that result in the release of elastic waves or acoustic emissions (AE). AE activity is monitored via two arrays of 8 piezoelectric transducers each, mounted inside the two steel piston rings at 45° intervals. Since the experiments lasted for up to three hours (with the slowest rate of rotation of 0.02 degrees/s) and because of the need to record AE waveforms at high temporal resolution (5 MHz), we set the data acquisition system to trigger mode. The system can be triggered by any of the 16 AE transducers. Every triggered event contains 16 waveforms, each with a duration of 5 ms. Pilot measurements had shown that the maximum duration of individual AE events did not exceed 3 ms. The trigger time stamps were used to calculate the waiting time between AE events. A trigger means some threshold value. To ensure that our interevent time statistics do not depend on this threshold, we checked that various values for the trigger threshold did not influence the reported statistics. The size of the event is derived from the maximum absolute amplitude ${A}_{i}$ of the 16 waveforms. We then define the size $S$ as $S={[median({A}_{1},\ldots ,{A}_{16})]}^{2}$.

### Numerical simulations

**Figure 9:**
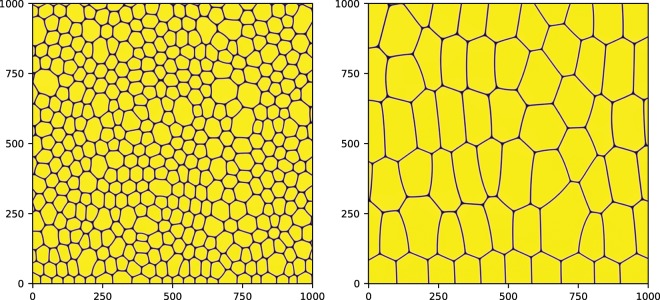
The left panel corresponds to the case of LBE size $L=1024$ and $R=0.09$. The right panel shows a 1024^2^ portion of the simulation performed with $L=4096$ and $R=0.03$.

**Figure 10:**
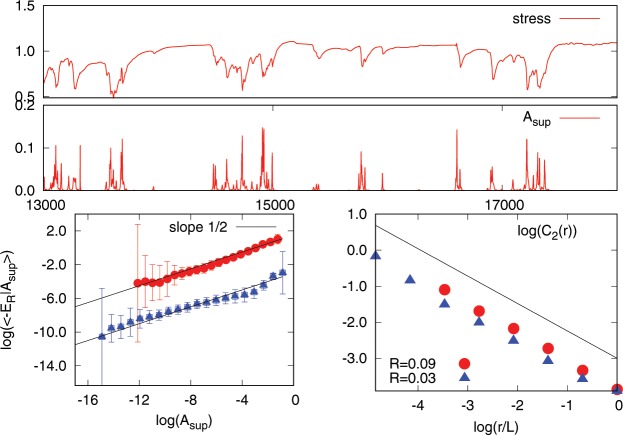
Upper two panels. Top: snapshot of the $xy$ component of the stress $\sigma$ as a function of time. Bottom: behavior of the quantity ${A}_{sup}(t)$, defined in the text, for the same time interval. Note that stress drops correspond to relatively large values of ${A}_{sup}$ due to irreversible lattice rearrangements. Lower two panels. Left: the average energy release ${E}_{r}$ conditioned on ${A}_{sup}$, see Eq. (4), as a function of ${A}_{sup}$ for two LBE simulations. Right: the scaling behavior of ${C}_{2}(r)$ as a function of $r$ for the two numerical simulations with $R=0.09$ (red bullets) and $R=0.03$ (blue triangles) corresponding to LBE grids 1024 and 4096 respectively.

The simulations use a model based on Lattice Boltzmann equations (LBE) for complex fluids^28^, which are discussed in detail in^29,39,40^. The model simulates two repelling fluids, say $A$ and $B$, with the same density. Coarsening is strongly suppressed by using a frustration mechanism, which stabilizes the interface. The initial configuration is chosen such that $N$ droplets of the fluid $A$ are randomly created in space with small polydispersity. The interface is filled by fluid $B$. The system exhibits a yield stress rheology with a non-Newtonian relation between stress and shear strain rate above the yield stress^29^. The ratio $R$ between the interface area ${A}_{int}$ and the bubble area ${A}_{b}$ can be estimated as

2 $$R\sim \frac{2\delta \sqrt{N}}{L}$$

where $\delta$ is the interface thickness, $N$ is the overall number of bubbles and $L$ is the size of the system in LBE units. Note that, qualitatively, the quantity $1-R$ can be considered as the packing fraction of the system. Since the interfaces are not sharp in our system, the correct packing fraction should be $1-CR$, with $C$ a constant of order $1$. Since $\delta$ must be finite for the interface to be stable, the only way to decrease $R$ (i.e. to increase the packing fraction) is to decrease the ratio $\sqrt{N}/L$. By decreasing $R$, we also increase the value of the yield stress, i.e. we increase the rigidity of our system. Examples of initial configurations with $R=0.09$ and $R=0.03$ are shown in Fig. 9a,b. The initial conditions $R=0.03$ together with $L=4096$ give a similar number of bubbles to that obtained for $R=0.09$ and $L=1024$. We will use results from both cases. The rheological properties of such systems are discussed in detail in^29^. We perform simulations with a small externally imposed shear strain rate, whose value is chosen such that the stress is below the yield stress transition. An example of the resulting stress $\sigma$ as a function of time is shown in Fig. 10 upper panel: a clear stick-slip behavior is observed. To perform a detailed statistical analysis of the dynamics, we used the method recently developed elsewhere^31^. We consider ${n}^{2}$ small squares of size $L/n$ and, for each of the squares, we compute the quantity ${A}_{i}\equiv \langle {({\rho }_{A}(x,y,t+\tau )-{\rho }_{A}(x,y,t))}^{2}\rangle$, where ${\rho }_{A}$ is the density of fluid $A$, $\langle \ldots \rangle$ is the space average over the square $i$ and $i=1,2,\ldots ,{n}^{2}$. We chose $n=32$ and $\tau =1000$ LBE time steps. We checked that different choices do not change the results discussed in the rest of this section. The quantity ${A}_{i}$ is a measure of the relative number of points in square $i$ which move in time interval $\tau$, i.e. ${A}_{i}$ is the square of the displacement occurring in the square $i$. Plastic events are localized in space and correspond to the largest value of ${A}_{i}$ observed in the system at time $t$. Therefore the relevant quantity to consider is^31^:

3 $${A}_{sup}(t)=su{p}_{i}[{A}_{i}(t)]$$

A large value of ${A}_{sup}(t)$ corresponds to a large stress drop in the system. In Fig. 10 we show in the upper panel $\sigma (t)$ and in the middle panel ${A}_{sup}(t)$ for the same time windows. To make a more quantitative analysis, we computed the quantity

4 $${E}_{r}=[-\sigma \frac{d\sigma }{dt}|{A}_{{\sup }}]$$

where ${E}_{r}$ represents the time averaged value of the energy release −$\sigma d\sigma /dt$ conditioned on a particular value of ${A}_{sup}$. In the lower left panel of Fig. 10 we show ${E}_{r}$ as a function of ${A}_{sup}$ for the two simulations at $R=0.09$ (red circles) and $R=0.03$ (blue triangles). A clear scaling law with a slope of 1/2 is observed. Using this result, we can state that the following relation holds scaling wise

5 $${E}_{r}\sim {A}_{sup}^{\mathrm{1/2}}$$

Next we investigated the spatial correlation in the system and computed the variables

6 $${\psi }_{r}\equiv \frac{1}{{r}^{2}}\,{\int }_{B(r)}\,dxdy{[\frac{{A}_{i}}{\langle A\rangle }]}^{2}$$

where $B(r)$ is a box of side $r$ and $\langle \ldots \rangle$ denotes the spacial average. Using ${\psi }_{r}$, we can construct the multi-fractal quantities ${C}_{q}(r)=E[\langle {\psi }_{r}^{q}\rangle ]$, where $E[\ldots ]$ is the time average. For a multi-fractal system we should observe ${C}_{q}(r)\sim {r}^{(q-\mathrm{1)(}{D}_{q}-d)}$, where the exponents ${D}_{q}$ are the generalized fractal dimensions and $d$ is the space dimension ($d=2$ in our case). Using the above definition of ${C}_{q}$, the space correlation of ${A}_{i}$ is associated with ${D}_{2}$ known as the correlation dimension, namely $E[\langle {A}_{i}{A}_{j}\rangle ]\sim {r}^{{D}_{2}-2}$ where ${A}_{i}$ ans ${A}_{j}$ are separated by the distance $r$. The red circles in the lower right panel of Fig. 10 corresponds to the quantity ${C}_{2}(r)$ computed from ${A}_{i}$. A clear scaling is observed with exponent ~−0.7 corresponding to ${D}_{2}\sim 1.3$. Therefore we can conclude that the system displays strong correlations in space. The same feature is observed using the overlap-overlap correlation function^39^.

**Figure 11:**
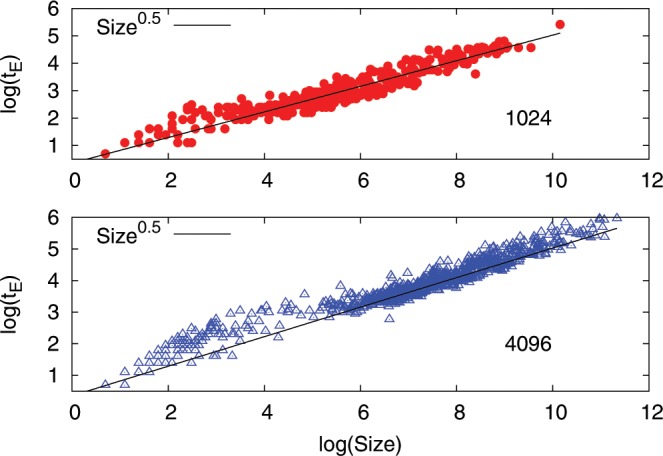
Upper panel: avalanche time duration ${t}_{E}$ versus avalanche size $S$ observed in LBE simulation $L=1024$, $R=0.09$. Bottom panel: the same as above but for LBE simulation $L=4096$, $R=0.03$. In both simulations, the duration time ${t}_{E}$ of the avalanche satisfies the scaling relation ${t}_{E}\sim {S}^{z}$ with $z\sim \mathrm{1/2}$.

We then analyzed the probability distribution of ${A}_{sup}$. In Fig. 2, we show $P({A}_{sup})$ as a function of ${A}_{sup}$ for $R=0.09$ and $R=0.03$. As already discussed elsewhere^31^, a clear scaling is observed for ${A}_{sup} > {A}_{th}$ over 2 decades, where ${A}_{th}$ is some threshold value (the same for both cases). The scaling exponent $P({A}_{sup})\sim {A}_{sup}^{-\gamma }$ is given by $\gamma \sim 1.33$. The quality of the scaling is best appreciated in the inset where we compensate for the power law, i.e we plot $P({A}_{sup})$/${A}_{sup}^{-1.33}$. The clear definition of ${A}_{th}$ appearing in the figure can be used to compute the scaling properties of the avalanche dynamics. We defined the time duration ${t}_{E}$ of the avalanche as the time interval when ${A}_{sup} > {A}_{th}$. Within each avalanche of duration ${t}_{E}$, we computed the size $S$ of the avalanche as the number of the ${n}^{2}$ region where ${A}_{i} > {A}_{th}$. The numerical simulations were performed for $40\times {10}^{6}$ LBE steps in the case of $R=0.09$ and for $280\times {10}^{6}$ LBE times steps for the case of $R=0.03$. A clear dynamical scaling ${t}_{E}\sim {S}^{z}$ is observed with $z\sim \mathrm{1/2}$, close to mean field predictions^2^, see Fig. 11. From this dynamical scaling we therefore obtain

7 $$S\sim {t}_{E}^{2}\sim {\int }_{{t}_{E}}\,dt[-\sigma \frac{d\sigma }{dt}]\sim {t}_{E}{E}_{r}\sim {t}_{E}{A}_{sup}^{\mathrm{1/2}}\sim {A}_{sup}$$

It follows that the scaling exponent $\gamma$ previously defined is also the scaling exponent of the probability distribution of $S$, i,e, $P(S)\sim {S}^{-1.33}$ similar to what has been found in many numerical simulations^10^.
